# Transcriptome and methylome sequencing reveals altered long non-coding RNA genes expression and their aberrant DNA methylation in equine sarcoids

**DOI:** 10.1007/s10142-023-01200-2

**Published:** 2023-08-08

**Authors:** Ewelina Semik-Gurgul, Artur Gurgul, Tomasz Szmatoła

**Affiliations:** 1 Department of Animal Molecular Biology, National Research Institute of Animal Production, Krakowska 1 St., 32-083 Krakow, Balice Poland; 2 Center for Experimental and Innovative Medicine, University of Agriculture in Krakow, Redzina 1c, 30-248 Krakow, Poland

**Keywords:** Equine sarcoid, Differentially expressed genes, DElncRNAs, DNA methylation, Long non-coding RNA, Transcriptome, Tumor

## Abstract

Recent publications confirmed that long non-coding RNAs (lncRNAs) perform an essential function in gene-specific transcription regulation. Nevertheless, despite its important role, lncRNA has not yet been described in equine sarcoids, the skin neoplasia of horses. Therefore, the aim of this study is to deepen the knowledge about lncRNA expression in the pathogenesis of equine sarcoids and provide new insight into the regulatory function of lncRNA in the bovine papillomavirus–dependent neoplasia of horse dermal tissues. RNA sequencing (RNA-seq) data from 12 equine sarcoid samples and the corresponding controls were reanalyzed in this study. A total of 3396 differentially expressed (DE) lncRNAs and 128 DElncRNA-DE genes (DEGs) pairs were identified. Differentially expressed lncRNAs predicted target genes were enriched in pathways associated with inter alia the extracellular matrix disassembly and cancer pathways. Furthermore, methylation data from the same samples were integrated into the analysis, and 12 DElncRNAs were described as potentially disturbed by aberrant methylation. In conclusion, this study presents novel data about lncRNA’s role in the pathogenesis of equine sarcoids.

**Supplementary Information:**

The online version contains supplementary material available at 10.1007/s10142-023-01200-2.

## Introduction

Equine sarcoids are horses’ most frequent skin tumors, but other equids, including zebras, donkeys, and mules, can also be affected (Knottenbelt 2005). Sarcoids are fibroblastic, locally invasive, nonmetastatic tumors that appear to develop as a result of the interaction of several factors, including bovine papillomavirus (BPV) infection, chronic physical trauma, altered wound healing, or genetic predisposition (Knottenbelt et al. 1995; Cochrane 1997; Hanson 2008). Due to their form, they contribute to the formation of secondary ulceration or infection and thus significantly impact the welfare and functioning of the affected animals (Semik-Gurgul 2021; Offer et al. 2023). Novel tools and strategies for effective diagnosis and treatment of horse sarcoid are constantly sought after. In recent years, it has been established that long noncoding RNAs (lncRNAs) play an important role in the occurrence and development of cancer. At the same time, the use of lncRNAs in the diagnosis and treatment of tumors, also those found in animals, is attracting more attention of researchers. Numerous studies are currently being conducted to screen for new carcinogenesis markers, by detecting lncRNAs that are aberrantly expressed in tumor cells (Beylerli et al. 2022). Therefore, the identification of lncRNA abnormal expression in horse sarcoids can broaden our knowledge about molecular mechanisms involved in tumorigenesis as well as could be used as the basis for developing novel alternative diagnostic and therapeutic approaches in their treatment.

Long non-coding RNAs (lncRNAs) are a class of ncRNAs with a length of >200 nucleotides, which cannot encode proteins but can act as gene expression modulators at the epigenetic, transcriptional, and post-transcriptional levels (Xia et al. 2022). The lncRNA transcription may negatively or positively control protein-coding gene expression and function through binding to histone-modifying complexes, to DNA-binding proteins, and even to RNA polymerase II (Long et al. 2017). A number of studies have shown that lncRNA participates in regulating various biological processes, such as genomic imprinting (Sleutels et al. 2002), X-chromosome inactivation (Zhao et al. 2008; Bischoff et al. 2013), and developmental processes (Paralkar et al. 2014; Zhao et al. 2015). Moreover, lncRNAs are also linked with disease processes, including cancer cell invasion, proliferation, apoptosis, differentiation, development, and metastasis (Iyer et al. 2015; Bhan et al. 2017; Rahman et al. 2021). In addition, available research results indicate that many lncRNAs show tissue-specific (TS) expression patterns, often restricted to a single cell line (Jiang et al. 2016), which may provide a new source for specific biomarkers for tumor cell identification.

It is well known that DNA methylation is one of the most important epigenetic mechanisms and key regulators of gene expression. The latest studies have discovered that lncRNAs with aberrant methylation patterns might be involved in cancer development and progression. It has been also suggested that lncRNAs showing aberrant DNA methylation may serve as potential epigenetically based diagnostic factors (Guo et al. 2018; Song et al. 2020). Therefore, investigation of the relationship between DNA methylation and lncRNA expression may be crucial to understanding the basics of equine sarcoids formation and identifying potential diagnostic markers.

Our previous study of equine sarcoids (Semik-Gurgul et al. 2023) determined their transcriptome by RNA sequencing (RNA-seq), but the analysis focused only on the protein-coding genes without considering the functions of lncRNA. However, long non-coding RNAs are emerging as an interacting factor in gene expression regulation. The present study, therefore, extends our transcriptomic analysis of the horse sarcoids into the category of differentially expressed lncRNAs (DElncRNAs). By re-analyzing our published RNA-seq datasets (GSE226986), we identified DElncRNAs, their correlated DEGs, and potential functional networks that contain these two classes of transcripts. Finally, we screened the DNA methylation sites located in the DElncRNAs promoter regions to analyze the factors that may affect the expression of identified DElncRNAs.

## Materials and methods

### Tissue samples

Samples of sarcoid tissues from 12 horses aged 4 to 22 years and belonging to the following breeds: Polish Half Bred Horse, Ponies, Oldenburg, and Thoroughbred, and 12 healthy skin samples (controls) from cold-blooded horses obtained at the slaughterhouse were the same as those used in our previous study (Semik-Gurgul et al. 2023). The tumors were histologically confirmed. In addition, the presence of BPV DNA was found in the analyzed lesion samples, and at the same time, its absence was confirmed in the control samples (Semik-Gurgul et al. 2023).

For validation study with qPCR, total RNA was isolated using TRIzol reagent (Invitrogen, Thermo Fisher Scientific, Waltham, MA, USA) in combination with a Direct-zol RNA kit (Zymo Research, Irvine, CA, USA). cDNA was synthesized from the same tissue samples that were used for RNA-seq using 400 ng of RNA and the High Capacity cDNA Reverse Transcription Kit (Thermo Fisher Scientific, Waltham, MA, USA). The procedures were carried out according to the manufacturer’s instructions.

### Data source

In this study, we used RNA-seq pair-end sequencing data from the abovementioned 12 sarcoid tissues and 12 healthy skin tissues from the GEO database GSE226986 series (Semik-Gurgul et al. 2023). The DNA methylation data obtained from the RRBS method (GSE208778; (Semik-Gurgul et al. 2023)) were derived from the same samples as the ones used for the RNA-seq data generation.

### Identification of differentially expressed lncRNAs and mRNAs

To identify DElncRNAs, a comprehensive reference list of known lncRNAs was included in the processing of the RNA-seq data. Briefly, FASTQ data were quality controlled, and reads were trimmed with FlexBar software (Dodt et al. 2012). The filtered reads files were mapped using STAR aligner software (Dobin et al. 2013), and mapped reads were counted using HTSeq-count software (Anders et al. 2015) using the Ensembl GTF annotation file of EquCab3.0 assembly release 109 that contains information on the 15,169 intergenic lncRNA and 21,468 coding genes. Differential expression analysis of lncRNAs (DElncRNAs) and genes (DEGs) was conducted using the DESeq2 R package (Love et al. 2014). The DElncRNAs and DEGs were selected with a cutoff of FDR<0.1 (Benjamini–Hochberg *p* value adjustment).

### DElncRNA genomic context analysis

The genomic context of DElncRNAs was determined in relation to protein-coding genes based on the protocol presented by Pang et al. (2009). Briefly, transcripts that were mapped head-head to the protein-coding gene at a distance of <1000 bp were defined as the bidirectional lncRNAs. Intergenic lncRNAs were defined as transcripts mapped within an intergenic region, without the presence of any overlapping or bidirectionally coded sequences for transcripts nearby.

### DElncRNA target gene prediction

To determine cis-target genes of the DElncRNAs in sarcoids, we searched for coding genes located 10-Kb upstream of the identified DElncRNAs and analyzed their functional roles. We computed Pearson’s rank correlation coefficient between each pair of DElncRNA– protein-coding genes. The protein-coding genes having significant correlations (*p*<0.05) with DElncRNAs at |*r*|>0.85 or |*r*|<−0.85 were considered potential cis-target genes for those DElncRNAs.

### Functional analysis of DElncRNAs target genes

The identified DElncRNA target genes were subjected to Gene Ontology (GO) functional term analysis and Kyoto Encyclopedia of Genes and Genomes (KEGG) signaling pathway enrichment analysis using ShinyGO software v 0.77 (Ge et al. 2020). All known genes were set as a background for enrichment, and obtained *p* values were corrected for multiple testing using the FDR procedure (Benjamini and Hochberg 1995).

### Integrative analysis of DNA methylation and lncRNA expression

We selected the methylation sites located within the promoter regions (1500bp upstream of the transcription start site—TSS1500) of differentially expressed lncRNA based on RRBS results from our previous data (GSE208778; (Semik-Gurgul et al. 2023)). The correlation analysis between the expression level and the corresponding DNA methylation sites of each DElncRNA was calculated, and those with *r*<−0.50 and *p*<0.05 were considered significant.

### Validation of DElncRNAs

Six DElncRNA were screened using quantitative reverse transcription PCR (qRT-PCR) to verify the reliability of the analysis. qRT-PCR was performed on the reversely transcribed RNA with the AmpliQ 5× HOT EvaGreen® qPCR Mix Plus (ROX) kit (Novazym, Poznan, Poland) and using Quant Studio 7 Flex (Thermo Fisher Scientific). Primers for mRNA sequences were designed to span two adjacent exons with Primer3 software (version 0.4.0) (https://bioinfo.ut.ee/primer3-0.4.0/) (Table S3A), and UBB and B2M genes (Bogaert et al. 2006) were used as endogenous control. Each sample was run in triplicate. The relative expression levels of each lncRNA were calculated using the ∆∆Ct method. The qRT-PCR results were subjected to statistical analysis using JASP software v. 0.16.3. Differences between relative expression values were tested by the Mann–Whitney or *t* test after distribution evaluation with the Shapiro–Wilk normality test (JASP Team, 2022).

## Results

### Basic characteristics of the lncRNAs

**Fig. 1:**
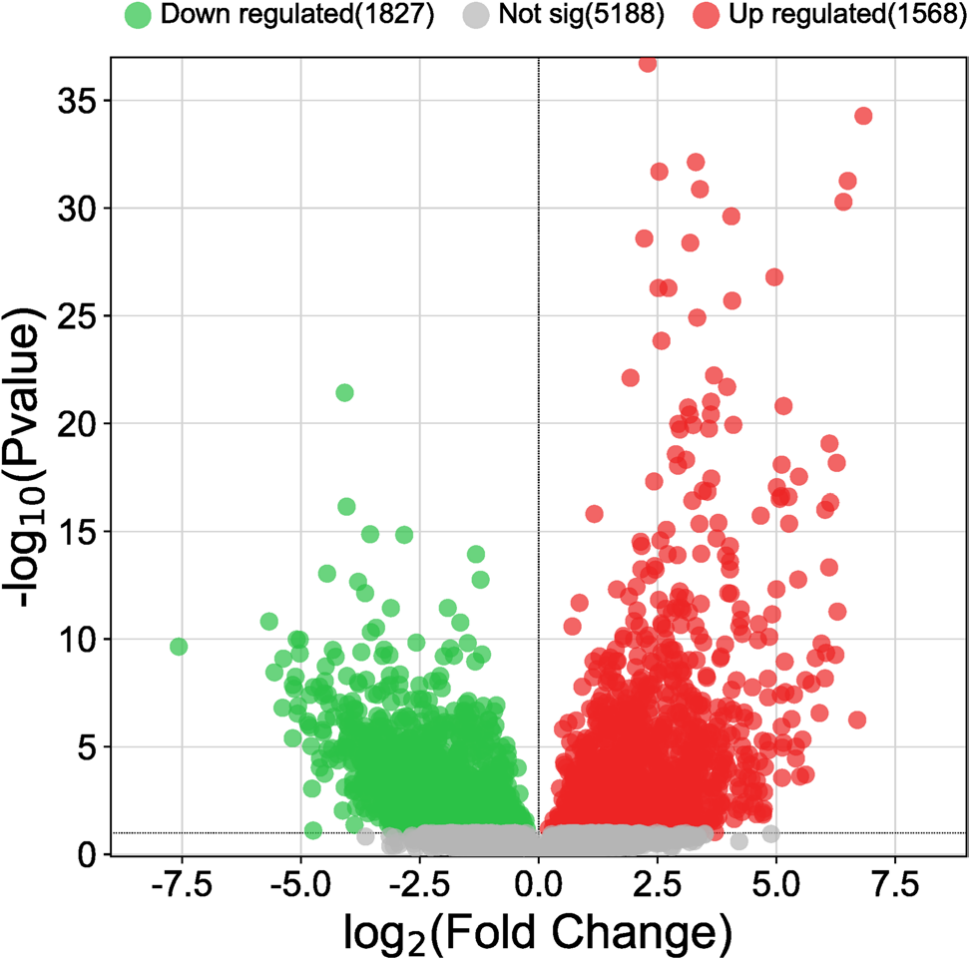
Volcano plot showing expression changes of long non-coding RNAs (lncRNAs) between sarcoid and control samples

In this study, lncRNA transcripts annotated in the ENSEMBL gtf annotation v109 have been used as a basis for comparative analysis. The set included 15,166 lncRNAs whose expression was retrieved for all samples (Fig. 1). Those lncRNAs ranged in size from 203 to more than 29,800 nucleotides (nt), and most of them (*N*=10,054; 66.3%) were medium size-lncRNA (950–4800 nt) followed by small-lncRNA (200–950 nt) (*N*=4115; 27.1%) and large-lncRNA (>4800 nt) (*N*=996; 6.6%,) (Table S1A). The distribution of lncRNAs across chromosomes was heterogeneous, and most of the lncRNAs exist in chromosome 1 (Chr1) (*N*=1215; 8.01%) followed by Chr2 (*N*=827) and Chr14 (*N*=680), and the average number of lncRNAs per chromosome was 459 ±216.35 SD (Table S1B). According to the position of lncRNAs with respect to adjacent coding genes, lncRNAs were mainly (*N*=14,381; 94.8%) intergenic, but also included bidirectional lncRNAs (*N*=785; 5.2%).

### Differentially expressed lncRNAs between sarcoids and control tissues

**Fig. 2:**
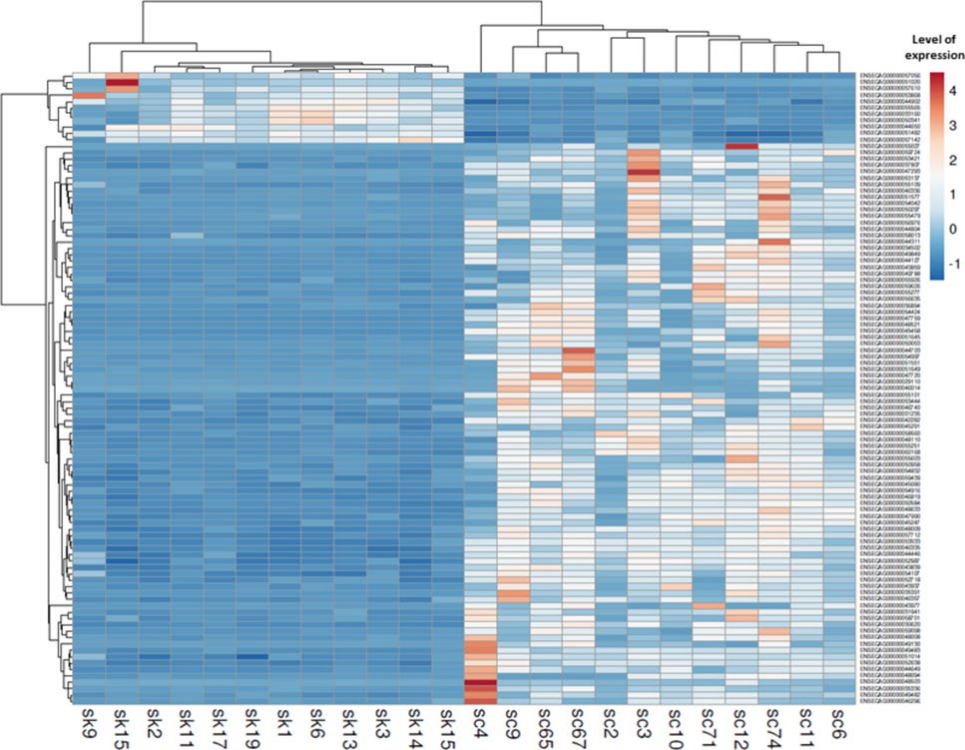
Clustering analysis of top 100 differentially expressed lncRNA between sarcoid and control samples. Rows are lncRNAs, and columns are samples (sc-sarcoid samples; sk-control samples). Blue color denotes downregulation in tumor samples and red denotes upregulation in lesional samples

Out of a total of 15,169 annotated long non-coding RNAs, 6960 (45.9%) lncRNAs were expressed in sarcoid and healthy tissues with a mean of normalized read counts higher than 1, and 3396 (22.4%) transcripts were significantly DE (FDR<0.1) between the two analyzed groups (sarcoids vs. control) (Fig. 2, Table S1C). In addition, 1569 were upregulated, and 1827 were downregulated in the tumor samples. Among the significant DElncRNAs, 2454 (72.3%) were medium-sized lncRNA, 541 (15.9%) were small-lncRNA, and 401 (11.8%) were classified as large-lncRNA. Table 1 presents the top 20 (10 upregulated and 10 downregulated) differentially expressed long non-coding RNAs in analyzed equine sarcoid samples.

**Table 1:** Top 20 (ten upregulated and ten downregulated) DElncRNAs (sorted by FDR value)

LncRNA	Position (chr:start–end)	Size (nt)	Log2FC*	FDR*
*ENSECAG00000047759*	chr6:62624294–62653328	4617	6.607	4.82E-57
*ENSECAG00000053533*	chr15:14942081–14943780	1608	2.292	1.92E-37
*ENSECAG00000048521*	chr6:62626129–62652907	1363	6.834	5.26E-35
*ENSECAG00000042282*	chr14:33955921–33992743	1143	3.305	7.36E-33
*ENSECAG00000052638*	chr15:14978387–15021280	1589	2.537	2.03E-32
*ENSECAG00000054424*	chr6:62611490–62615659	2012	6.503	5.46E-32
*ENSECAG00000055251*	chr27:25717287–25765099	3779	3.393	1.33E-31
*ENSECAG00000049849*	chr11:3785546–3787538	806	6.412	5.16E-31
*ENSECAG00000048633*	chr22:22149460–22155688	2106	4.053	2.42E-30
*ENSECAG00000046335*	chr15:14976423–14977963	822	2.222	2.60E-29
*ENSECAG00000033160*	chr20:7239878–7311487	18,189	−4.080	3.81E-22
*ENSECAG00000051020*	chr15:39409443–39412086	1081	−4.039	7.06E-17
*ENSECAG00000053868*	chr12:31664516–31685948	11,154	−3.544	1.35E-15
*ENSECAG00000057610*	chr11:2979996–2999261	4546	−2.826	1.45E-15
*ENSECAG00000051482*	chr2:46605755–46608450	2696	−1.318	1.15E-14
*ENSECAG00000055565*	chr20:7301866–7304258	1743	−4.449	9.05E-14
*ENSECAG00000044902*	chr7:5065253–5067191	1838	−1.223	1.75E-13
*ENSECAG00000050341*	chr20:7280980–7282824	921	−3.797	2.19E-13
*ENSECAG00000044650*	chr11:25786610–25801437	3488	−3.651	7.41E-13
*ENSECAG00000057142*	chr3:29801642–29806895	4683	−1.913	3.61E-12

*Log2FC Logarithm of fold change; *FDR* false discovery rate

### Validation of the sequencing data by qRT-PCR

**Fig. 3:**
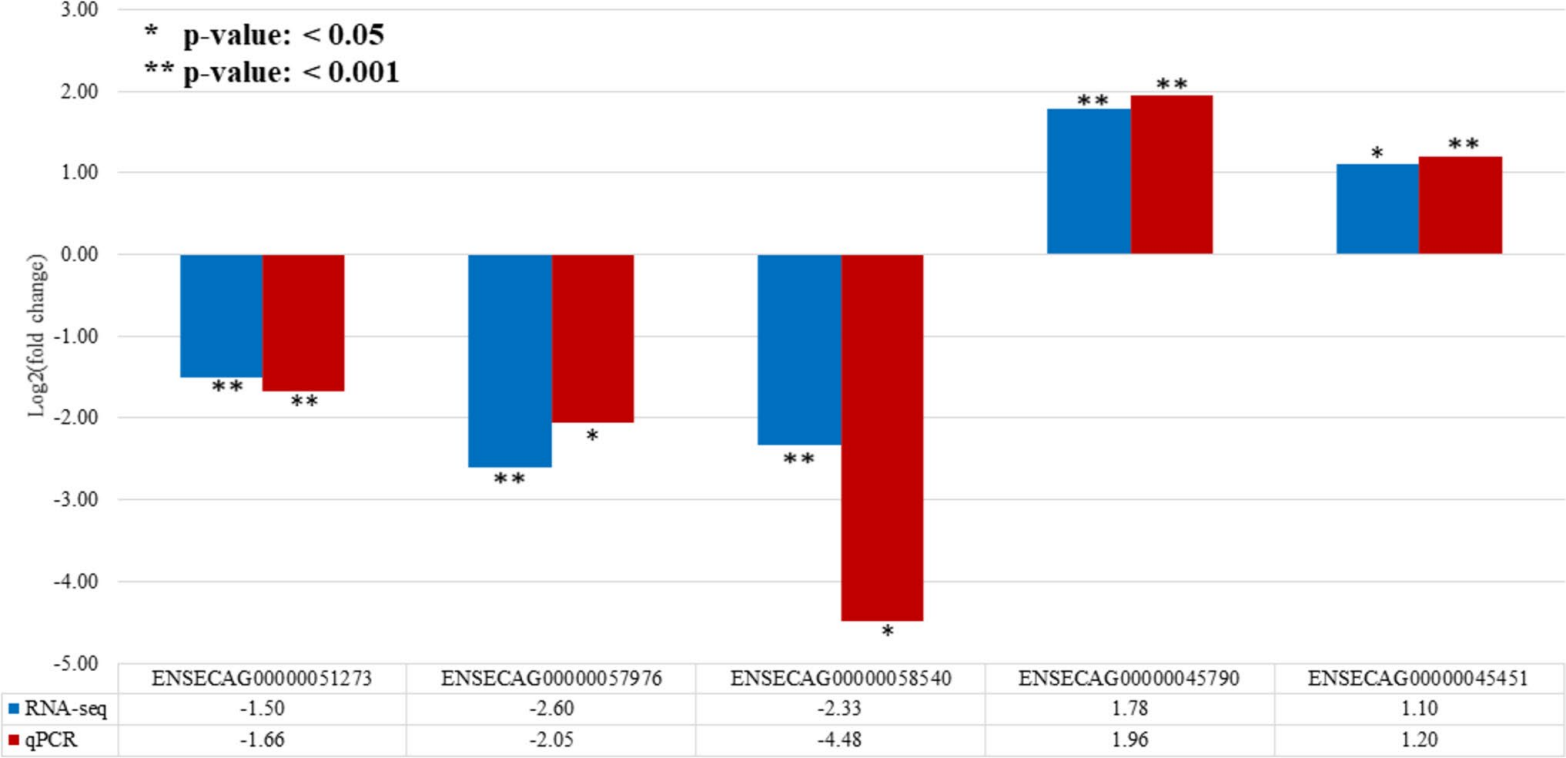
Validation of the differentially expressed lncRNAs in sarcoid samples by qRT-PCR and their comparison with results of RNA sequencing

To validate the sequencing data, we investigated the expression pattern of five randomly selected lncRNAs, which were identified to be differentially expressed in tumor tissue. The validation of the level of gene expression showed significant differences (|logFC| 1.20–4.48; *p*<0.05) between the studied groups (Fig. 3, Table S3B). The correlation coefficient between qRT-PCR values and data from high-throughput sequencing was positive and ranged from 0.433 to 0.846 (*p*<0.05), confirming the high accuracy of the transcriptomic results.

### Correlations between DElncRNAs and the expression of DEGs

In order to analyze the potential function of the differentially expressed lncRNAs in horse sarcoids, we studied the interaction between the differently expressed lncRNA and 10,512 identified differently expressed protein-coding genes (FDR<0.1). A total of 128 DElncRNA-DEG pairs were identified, based on the threshold of significant correlation coefficient >|0.85| (Table 2, Table S1D). The obtained values of negative correlations were low and ranged from −0.02 to −0.73. Among the identified DElncRNA-DEG pairs, four DElncRNAs were paired with more than one DEG. Specifically, *ENSECAG00000057142* were paired with *GAN* and *ENSECAG00000033092*, *ENSECAG00000051273* with *PPARA* and *ENSECAG00000031323*, *ENSECAG00000046304* with *ADGRL3* and *ENSECAG00000031668*, and finally *ENSECAG00000046010* with *ABI2* and *CYP2-A1* genes. Furthermore, the group of potential target genes were identified DEGs encoding proteins belonging to the collagen (*COL14A1*) and ADAMTS families (*ADAMTS2*, *ADAMTS15*), transcription factors (*SOX7*, *TP73*, *TFAP2C*), or cell adhesion molecules (*CADM2*, *NECTIN3*) (Table S1D).

**Table 2:** Top ten identified DElncRNA-DEG pairs (sorted by correlation coefficient value)

DElncRNA	Log2FC*	DEG [gene symbol]	Log2FC*	Correlation value
*ENSECAG00000051025*	3.98	*ENSECAG00000003099 [MGRG1]*	2.45	0.994
*ENSECAG00000045790*	1.78	*ENSECAG00000017743 [SESN3]*	1.65	0.994
*ENSECAG00000048094*	4.02	*ENSECAG00000011901 [UNC5D]*	3.91	0.993
*ENSECAG00000056686*	3.20	*ENSECAG00000014254 [LCTL]*	2.95	0.993
*ENSECAG00000045951*	−3.31	*ENSECAG00000000267 [SLC5A1]*	−3.33	0.992
*ENSECAG00000057142*	−1.91	*ENSECAG00000012155 [GAN]*	−1.94	0.991
		*ENSECAG00000033092*	−2.19	0.991
*ENSECAG00000048455*	1.62	*ENSECAG00000015715 [ADAMTS15]*	1.55	0.989
*ENSECAG00000045415*	−0.80	*ENSECAG00000020208 [LIFR]*	−0.80	0.989
*ENSECAG00000049467*	0.78	*ENSECAG00000012918 [ZNF704]*	0.67	0.986

*Log2FC Logarithm of fold change

### GO enrichment and KEGG pathway analysis of DElncRNA targets genes

The 128 candidate target genes were subjected to GO and pathway analyses. Functional analysis showed that target genes of DElncRNAs were significantly enriched (FDR<0.1) in 321 GO terms, including 283 biological process (BP) terms, 17 cellular component (CC) terms, and 21 molecular function (MF) terms. Among the top twenty biological terms related to the differentially expressed lncRNAs in horse sarcoids were processes related to the regulation of Ras protein signal transduction (GO:0046578; FDR=0.004), positive regulation of nucleic acid–templated transcription (GO:1903508; FDR=0.002) or cell population proliferation (GO:0008283; FDR=0.006). Other detected GO terms were related to the apoptotic process (GO:0006915; FDR=0.02), negative regulation of cell death (GO:0060548; FDR=0.07), epithelial cell morphogenesis (GO:0003382; FDR=0.07), or extracellular matrix disassembly (GO:0022617; FDR=0.08). Significantly enriched cellular components included inter alia receptor complex (GO:0043235; FDR=0.007), an integral component of postsynaptic density membrane (GO:0099061; FDR=0.02), and cell junction (GO:0030054; FDR=0.03). They were also linked to molecular functions such as transcription cis-regulatory region binding (GO:0000976; FDR=0.008), transcription regulator activity (GO:0140110; FDR=0.03), or transcription factor binding (GO:0008134; FDR=0.07) (Table S2A-C).

**Fig. 4:**
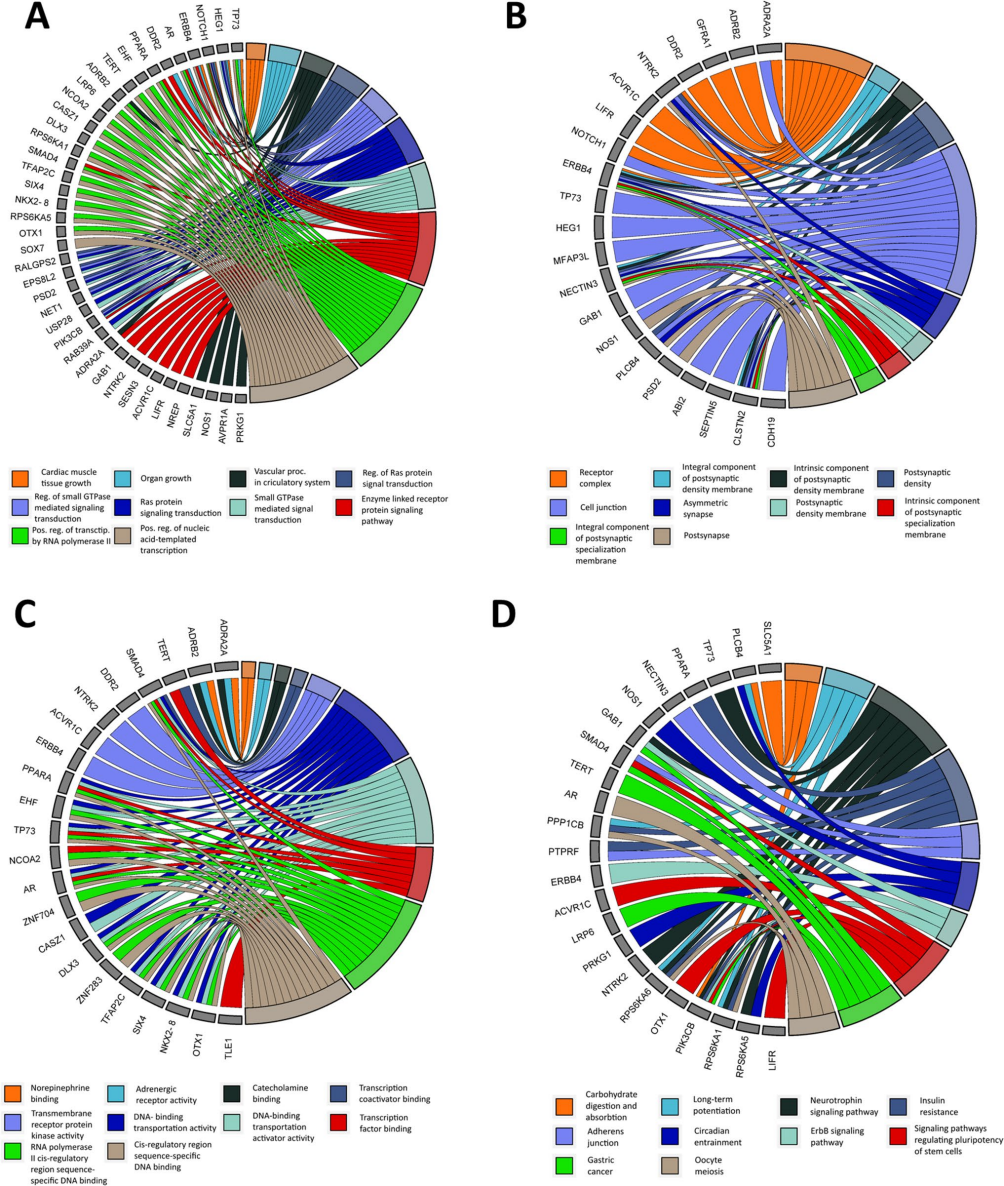
Classification of DElncRNA target genes to top ten the most overrepresented annotation categories **A** GO biological processes. **B** GO cellular components. **C** GO molecular functions. **D** KEGG signaling pathways

The potential target genes of differentially expressed lncRNAs were also subjected to the Kyoto Encyclopedia of Genes and Genomes (KEGG) signaling pathway enrichment analysis. The results revealed 24 significantly enriched (FDR<0.1) pathways. Among them, there were those linked with diseases such as cancer (ecb05200, FDR=0.07), chemical carcinogenesis (ecb05207, FDR=0.05), or hepatocellular carcinoma (ecb05225, FDR=0.04). In addition, significantly enriched pathways included also MAPK signaling pathway (ecb04010, FDR=0.06) or ErbB signaling pathway (ecb04012, FDR=0.07) involved in various cellular functions, including cell proliferation, differentiation, and migration (Fig. 4A–D, Table 3, Table S2D).

**Table 3:** Top 20 enriched KEGG pathways involving DElncRNA targets genes (sorted by enrichment FDR)

Pathway	Enrichment FDR	nGenes*	Pathway genes*	Fold enrichment
Neurotrophin signaling pathway	5.05E-04	7	119	11.39
Insulin resistance	1.95E-03	6	109	10.66
Long-term potentiation	2.14E-02	4	65	11.92
Signaling pathways regulating pluripotency of stem cells	3.28E-02	5	140	6.92
Gastric cancer	3.28E-02	5	144	6.72
Circadian entrainment	3.99E-02	4	96	8.07
Hepatocellular carcinoma	3.99E-02	5	159	6.09
Carbohydrate digestion and absorption	4.14E-02	3	47	12.36
Chemical carcinogenesis	4.99E-02	5	181	5.35
Oocyte meiosis	5.08E-02	4	118	6.56
Platelet activation	5.08E-02	4	119	6.51
Thyroid hormone signaling pathway	5.08E-02	4	119	6.51
MAPK signaling pathway	5.68E-02	6	292	3.98
Vascular smooth muscle contraction	6.30E-02	4	132	5.87
Adherens junction	6.68E-02	3	71	8.18
Pathways in cancer	6.68E-02	8	524	2.96
ErbB signaling pathway	7.48E-02	3	84	6.92
Calcium signaling pathway	7.48E-02	5	235	4.12
CGMP-PKG signaling pathway	7.48E-02	4	160	4.84
Phospholipase D signaling pathway	7.48E-02	4	154	5.03

*nGenes The number of target genes assigned to a given pathway; *Pathway Genes* total genes in pathway

### Integrated analysis of DNA methylation and DElncRNAs expression

To explore the DNA methylation pattern of lncRNAs in sarcoids, we compared the methylation level of RRBS-generated CpG sites in 12 tumors and 12 control tissues previously used for the lncRNA analysis. A total of 1989 differentially methylated sites (DMSs) with a cutoff value of at least 25% methylation difference between the two groups and a *q* value of <0.05 were obtained in the promoters and within the gene body of lncRNAs (Table S1E). Among the identified DMSs, hypomethylation was observed for 57.97% (*N*=1153) of all DMSs and was higher than the number of hypermethylated CpGs (*N*=836; 42.03%). The annotation of DMSs according to lncRNA features revealed that most DMSs were located in introns (65%), followed by those in exon regions (18.2%). Approximately 9.5% of DMSs were located in downstream regions, while 7.3% were found in the regions around the lncRNA transcription start site (TSS1500). The two omics data (methylome and transcriptome) were combined for further analysis. By associating the 1989 CpG sites with 3396 DElncRNAs, 23 pairs of potentially methylation-dependent DElncRNAs were identified, having DMSs in the promotor region, of which 12 were characterized with significant negative correlation value *r*<−0.50 between methylation and expression. Within those 12 pairs, five DElncRNAs showed decreased methylation levels in promotor regions and higher expression values, and seven were characterized by hypermethylated DMSs and lowered expression levels in sarcoid samples (Table 4). The two DElncRNAs with identified DMSs in the previous analysis (the “Correlations between DElncRNAs and the expression of DEGs” section) were paired with DEGs as their potential target genes. Namely, *ENSECAG00000047707* encompassing hypermethylated CpGs was paired with *CASZ1* and *ENSECAG00000057245* with detected hypomethylated DMS with *SRGAP3* genes.

**Table 4:** DElncRNAs with significant changes in expression and their DNA methylation levels in sarcoid samples

DElncRNA	CpG site location	Changes in DNA methylation level (%)	Changes in expression level (log2FC)	Correlation value*	Correlation *p* value
*ENSECAG00000056702*	Promoter	25.37	−2.11	−0.50	0.014
*ENSECAG00000059206*	Promoter	32.30	−1.47	−0.53	0.0115
*ENSECAG00000046149*	Promoter	34.05	−1.73	−0.50	0.016
*ENSECAG00000045198*	Promoter	29.71	−2.71	−0.62	0.002
*ENSECAG00000051120*	Promoter	47.74	−1.52	−0.64	0.001
*ENSECAG00000052555*	Promoter	36.62	−2.49	−0.51	0.013
*ENSECAG00000047261*	Promoter	28.57	−2.00	−0.86	<001
*ENSECAG00000052860*	Promoter	−27.44	0.83	−0.50	0.014
*ENSECAG00000049428*	Promoter	−26.36	1.54	−0.68	<001
*ENSECAG00000044152*	Promoter	−35.07	1.29	−0.67	<001
*ENSECAG00000053220*	Promoter	−33.27	3.44	−0.52	0.012
*ENSECAG00000051733*	Promoter	−27.62	1.62	−0.64	<001

*Correlation between changes in expression and methylation level

## Discussion

Sarcoids are known to be the most common skin tumor affecting equid health worldwide, and their underlying mechanism is still not fully understood. In this study, RNA-Seq data were used to analyze the changes in transcriptomic expression profiles of lncRNA in horse sarcoid samples. What’s more, the potential biological functions of the identified differentially expressed lncRNA were inferred by identifying the functional importance of adjacent protein-coding genes.

Until today, there was a lot of evidence supporting the hypothesis that dysregulated lncRNA expression may be involved in tumorigenesis and tumor progression (Gibb et al. 2011; Bartonicek et al. 2016; Qian et al. 2020). In our study, we conducted an analysis of the data from high-throughput sequencing to identify potential DElncRNAs interrelated with the formation and progression of equine sarcoids. To the best of our knowledge, this is the first report of analysis of the expression profiles of lncRNAs in equine sarcoid samples. By reanalyzing the previously generated sequencing data, we obtained information on the expression of 15,169 lncRNA transcripts that are annotated in the newest currently available ENSEMBL database (v.109). The results of transcriptome sequencing revealed that compared with the expression profiles of control tissue samples, there were 1569 upregulated and 1827 downregulated lncRNAs (FDR<0.1) in the tumor group.

This study also allowed the prediction of 128 potential targets/coexpressed genes for 125 DElncRNAs that were differentially expressed in tumor samples. The results of earlier research reveal that lncRNAs are coexpressed with adjacent or neighboring protein-coding genes (Cabili et al. 2011; Werner and Ruthenburg 2015; Núñez-Martínez and Recillas-Targa 2022). While looking for genes potentially significant for sarcoid progression, in the group of potential target genes we found inter alia *ADAMTS15*, *ADAMTS2*, and *COL14A1* genes. An aberrant expression of *ADAMTS* and collagen gene families has been observed in some pathological conditions, including cancer, and has been related to both oncogenic and tumor-protective roles (Rocks et al. 2006; Cal and López-Otín 2015; Xu et al. 2019). Recent reports suggest that *ADAMTS2* is overexpressed by gastric cancer cells, *COL14A1* is downregulated in breast cancer cells, and *ADAMTS15* functions as a tumor suppressor role in prostate cancer (Jiang et al. 2019; Binder et al. 2020; Malvia et al. 2023). Even if the relationship between these proteins and the formation of equine sarcoids remains unclear, it is conceivable (as shown for matrix metalloproteinases—MMPs) that these proteins contribute to the remodeling and degradation of the extracellular matrix (ECM)—one of the factors linked to the processes of tumor initiation and progression. The ECM is an intricate network that constantly undergoes remodeling and serves diverse functions in cell proliferation, adhesion, migration, polarity, differentiation, and apoptosis (Lu et al. 2011; Yue 2014). Recent studies have linked several lncRNAs with ECM remodeling processes within the tumor microenvironment. LncRNAs have been shown to regulate the expression of several ECM-associated molecules. Furthermore, the expression of dysregulated lncRNAs is closely correlated with that of ECM genes (Huang et al. 2016; D’Angelo and Agostini 2018; Akbari Dilmaghnai et al. 2021). For example, it was reported that the expression level of lncRNA H19 was significantly downregulated in the metastatic prostate cancer cell line and negatively correlated with the expression of the extracellular matrix protein *TGFBI* (Zhu et al. 2014). Previous studies demonstrated that an imbalance of the ECM and an abnormal expression of its genes play a major role in sarcoid pathogenesis (Martano et al. 2016; Podstawski et al. 2022). It is therefore of interest, that our study showed upregulation of three lncRNAs — *ENSECAG00000046740*, *ENSECAG00000058013*, and *ENSECAG00000048455* that may be connected with aberrant expression of their correlated genes, and thus indirectly affect the stability of the extracellular matrix during sarcoid formation and progression.

To validate the sequencing data, we randomly selected five different lncRNAs and performed quantitative reverse transcription PCR. The qRT-PCR results of analyzed lncRNAs are consistent with the sequencing data. The correlation coefficient between the two methods was positive and significant, confirming the reliability of the high-throughput sequencing results.

To further explore the regulatory roles of lncRNA expression changes during sarcoid tumorigenesis, we performed the analysis with Gene Ontology for the predicted lncRNA target genes. Pathway analysis showed that 321 significant pathways (FDR<0.1) involving the target genes of DElncRNAs were enriched, among them some are known to play an important role in the tumor cell biology, such as programmed cell death, apoptotic process, or cell migration. We also found that predicted target genes in GO analysis were significantly associated with the extracellular matrix disassembly pathway. As we previously mentioned the changes in ECM composition are reported to play a critical role in the development of horse sarcoids (Martano et al. 2016; Podstawski et al. 2022). These results indicated that lncRNAs may play certain roles in the expression of ECM-related genes and play important roles in the pathogenesis of equine sarcoids. What’s more, the KEGG pathway analysis showed that the target genes were involved in the cancer pathway, chemical carcinogenesis, or hepatocellular carcinoma which further supports the hypothesis that the abovementioned DElncRNAs and their target genes could be involved in the BPV-dependent neoplasia of equine dermal tissues via regulating various important pathways.

Finally, we also performed comparative analyses between differential DNA methylation and DElncRNA expression patterns between sarcoid and normal tissues. We thus identified 12 differentially expressed lncRNAs that could be regulated by aberrant DNA methylation. Epigenetic mechanisms, including DNA methylation, play a key role in the control of gene expression, and the alterations of the epigenetic modification are central events in tumor initiation and progression (Flavahan et al. 2017). To date, both hypermethylation and hypomethylation have been identified in all types of cancer cells (Ducasse and Brown 2006; Flavahan et al. 2017). What’s more, it has been described that dysregulated DNA methylation is also observed in the case of equine sarcoids (Semik et al. 2018; Semik-Gurgul et al. 2018a, b, 2023; Semik-Gurgul 2021; Pawlina-Tyszko et al. 2022). In the available literature, you can find a growing number of studies, the results of which confirm the identification of abnormal DNA methylation in specific lncRNA regions and its impact on tumor progression (Song et al. 2020, 2022; He et al. 2021; Recalde et al. 2022). In this regard, in previous work, authors reported hypermethylated lncRNA (*LIFR-AS1*) that was downregulated and associated with tumorigenesis, metastasis, and poor prognosis in colorectal cancer (CRC) (Song et al. 2022). Guo et al. (2018) identified aberrant hypermethylation of the regions around the transcription start site of the lncRNA *C5orf66-AS1* that was associated with its expression and was gastric cardia adenocarcinoma–specific. Our research showed that lncRNAs were majority hypomethylated, which was consistent with the previous observations that confirmed in equine sarcoid cells and tissue a lower DNA methylation level (Potocki et al. 2012; Semik et al. 2018; Semik-Gurgul et al. 2023). Moreover, by associating the identified DMSs and DElncRNas, we found 12 pairs of methylation-driven lncRNAs with significantly negative correlation between methylation and expression levels. It is consistent with the understanding that DNA methylation inhibits gene expression (Moore et al. 2012). Therefore, we considered them as lncRNAs potentially dysregulated by aberrant methylation modifications during the pathogenesis of equine sarcoids. In sarcoid, we also identified CpG sites hypermethylation within *ENSECAG00000047707* lncRNA, and its expression level was significantly decreased in lesional samples. Recent studies have shown that changes in the expression of lncRNA mediated by alteration of its DNA methylation can further influence their gene targets (Song et al. 2020). The conducted comprehensive analysis of the lncRNA and mRNA expression profiles in sarcoid and control samples predicted inter alia the *CASZ1* gene as a potential target of this differentially expressed lncRNA. Interestingly, this gene was also characterized by reduced expression in the lesional samples. Based on these results, it can be speculated that abnormal expression of the *CASZ1* gene may be associated with the detected changes in DNA methylation of *ENSECAG00000047707*; however, this statement requires thorough confirmation in further research.

We understand that there are some limitations in this study. First, the annotation datasets that had been availed here to analyze long noncoding RNAs were adopted from the Ensembl database, in which only intergenic lncRNAs are annotated, and thus, some possibly important lncRNAs located within genes may be lacking from the current results. Second, the present study investigates only the putative cis-acting targets of lncRNAs in sarcoids, and further analysis should be performed to determine their trans-regulatory functions. The results of the present study are preliminary and primarily derived from bioinformatics analysis, so further experiments might be needed to validate our findings.

## Conclusions

In this study, we investigated potential lncRNAs interrelated with equine sarcoids using RNA-Seq and RRBS data sets. We preliminarily predicted the functions of DElncRNAs in lesional samples based on GO and KEGG function enrichment analysis of potential target genes of these lncRNAs. The present research revealed three differentially expressed lncRNAs that may participate in the development of horse sarcoids by their interactions with *ADAMTS2*, *ADAMTS15*, and *COL14A1* genes and indirectly affect the stability and remodeling changes of the extracellular matrix. Finally, we identified a set of lncRNAs whose expression is potentially disturbed during the process of tumorigenesis by DNA methylation. The obtained results provide a new example of the complexity and interdependence of various mechanisms involved in the regulation of gene expression in the process of equine sarcoid formation and progression. Further studies should be performed to determine the interactions between lncRNAs and the mentioned above target genes. Clarification of the precise transcriptional regulatory role of lncRNAs in horse sarcoid may help to understand the pathogenesis of this disease and facilitate the diagnosis and development of new therapies for this tumor.

## Supplementary information

Table S1 Statistics on long non-coding RNAs (lncRNAs): **(A)** Basic information on lncRNAs in the analyzed dataset. **(B)** Chromosomal distribution of lncRNAs. **(C)** Differentially expressed long non-coding RNAs (DElncRNAs) along with DE analysis statistics. **(D)** The list of DElncRNAs and their potential target genes. **(E)** The list of DMSs in different regions of lncRNAs. (XLSX 1118 kb)

Table S2 Functional enrichment analysis of DElnRNAs target genes: **(A)** The list of GO Biological Processes. **(B)** The list of GO Cellular Components. **(C)** The list of GO Molecular Functions. **(D)** The list of KEGG signaling pathways. (XLSX 16252 kb)

Table S3 The real-time qPCR analysis: (A) Primer sequences for real-time qPCR. (B) The results of validation of selected DElncRNAs with real-time qPCR. (XLSX 16 kb)

## Data Availability

The data used and analyzed during the current study may be viewed at https://www.ncbi.nlm.nih.gov/geo/query/acc.cgi?acc=GSE226986 and https://www.ncbi.nlm.nih.gov/geo/query/acc.cgi?acc=GSE208778.
